# PIAS3 expression in squamous cell lung cancer is low and predicts overall survival

**DOI:** 10.1002/cam4.372

**Published:** 2015-01-09

**Authors:** Rime Abbas, Karen S McColl, Adam Kresak, Michael Yang, Yanwen Chen, Pingfu Fu, Gary Wildey, Afshin Dowlati

**Affiliations:** 1 Division of Pulmonary and Critical Care Medicine, University Hospitals Medical Center, Case Western Reserve UniversityCleveland, Ohio, 44106; 2 Division of Hematology and Oncology, University Hospitals Medical Center and Seidman Cancer Center and Case Comprehensive Cancer Center, Case Western Reserve UniversityCleveland, Ohio, 44106; 3 Division of Pathology, University Hospitals Medical Center and Case Comprehensive Cancer Center, Case Western Reserve UniversityCleveland, Ohio, 44106; 4 Division of Biostatistics, Case Comprehensive Cancer Center, Case Western Reserve UniversityCleveland, Ohio, 44106

**Keywords:** PIAS3, squamous cell lung cancer, STAT3, survival

## Abstract

Unlike lung adenocarcinoma, little progress has been made in the treatment of squamous cell lung carcinoma (SCC). The Cancer Genome Atlas (TCGA) has recently reported that receptor tyrosine kinase signaling pathways are altered in 26% of SCC tumors, validating the importance of downstream Signal Transducers and Activators of Transcription 3 (STAT3) activity as a prime therapeutic target in this cancer. In the present report we examine the status of an endogenous inhibitor of STAT3, called Protein Inhibitor of Activated STAT3 (PIAS3), in SCC and its potential role in this disease. We examine *PIAS3* expression in SCC tumors and cell lines by immunohistochemistry of a tissue microarray and western blotting. *PIAS3* mRNA expression and survival data are analyzed in the TCGA data set. SCC cell lines are treated with curcumin to regulate PIAS3 expression and cell growth. PIAS3 protein expression is decreased in a majority of lung SCC tumors and cell lines. Analysis of *PIAS3* mRNA transcript levels demonstrated that low *PIAS3* levels predicted poor survival; Cox regression analysis revealed a hazard ratio of 0.57 (95% CI: 0.37–0.87), indicating a decrease in the risk of death by 43% for every unit elevation in *PIAS3* gene expression. Curcumin treatment increased endogenous PIAS3 expression and decreased cell growth and viability in Calu-1 cells, a model of SCC. Our results implicate PIAS3 loss in the pathology of lung SCC and raise the therapeutic possibility of upregulating PIAS3 expression as a single target that can suppress signaling from the multiple receptor tyrosine kinase receptors found to be amplified in SCC.

## Introduction

Lung cancer is the leading cause of cancer death in the United States, estimated at ∽160,000 deaths in 2014 1. Until the mid-1980's, squamous cell carcinoma occurred at the highest incidence among all lung tumors, a result of its strong association with cigarette smoking, which remains the leading risk factor for lung cancer development 2. While progress has evolved in the treatment of another non-small lung cancer variant, adenocarcinoma of the lung 3–6, with revelations on the genomics of this disease, effective targeted therapy in squamous cell lung cancer is still in its infancy 2,7,8.

Signal Transducers and Activators of Transcription 3 (STAT3) is a cytoplasmic transcription factor that is activated by tyrosine phosphorylation, leading to dimerization and translocation into the nucleus 9. In many cancers, constitutively activated STAT3 is associated with uncontrolled growth signaling and thus plays an essential role in tumorigenesis and cell proliferation. As a result, blockade of STAT3 appears to be an attractive target for cancer therapy, especially as it is activated by a number of upstream tyrosine kinases, including EGFR and FGFR1, and thereby serves as a central integration point for multiple oncogenic signaling pathways controlling cell cycle, apoptosis, angiogenesis, tumor invasion, and metastasis 10,11.

Multiple endogenous inhibitors of STAT3 exist, including the protein inhibitor of activated STAT3 (PIAS3) 12,13. A study by Brantley et al. 14 showed an absence of PIAS3 protein expression in glioblastoma multiforme, and demonstrated growth inhibition in U251 human glioblastoma cells when PIAS3 levels were restored. Similarly, we previously demonstrated 15 that two-thirds of squamous cell lung cancer human specimens displayed low to undetectable levels of PIAS3 by immunohistochemistry.

In the present study we build on this work by quantifying PIAS3 expression in squamous lung cancer tumors and cell lines by western blotting, determine its clinical correlation with survival using The Cancer Genome Atlas (TCGA) cohort of squamous lung cancer patients, and demonstrate upregulation of endogenous PIAS3 expression by curcumin, providing proof-in-principal that PIAS3 can be a potential target in lung cancer therapy.

## Materials and Methods

### Cell culture

Human pulmonary benign and malignant epithelial cell lines, NL-20 and A549 respectively, as well as human squamous cell lung cancer cell lines Calu-1, H520, H1869, H1385, and SW900 were purchased from American Type Culture Collection (Manassas, VA) and maintained in Dulbecco's Modified Eagle Medium (DMEM)/Ham's F-12 medium supplemented with 10% (v/v) fetal bovine serum (Hyclone; ThermoFisher Scientific, Waltham, MA) and 2 mmol/L l-Glutamine (GlutaMAX; Invitrogen, Camirillo, CA) in a 5% CO_2_ humidified incubator at 37°C.

### Curcumin treatment

SW900*,* Calu-1, and H520 cells were treated with increasing concentrations of curcumin (EMD Millipore, Billerica, MA) for 24 h after which cells were collected for immunoblotting protein lysates. Calu-1 cells were also treated with 5 *μ*mol/L curcumin for various times up to 72 h.

### Immunoblotting

Cell line lysates were prepared in RIPA buffer as previously described 15,16. Protein pellets of surgically resected human squamous cell lung cancer specimens were obtained from the Lung Cancer Biospecimen Resource Network (LCBRN, University of Virginia). The dried protein precipitate pellet was suspended in buffer containing 5% SDS, 1% Triton, 50 mmol/L Tris, and 150 mmol/L sodium chloride containing a Complete Mini Protease Inhibitor Tablet (Roche, Indianapolis, IA) and sonicated for 7–10 sec on ice. Protein was quantified by the Pierce BCA protein assay kit (ThermoFisher Scientific). All protein samples were electrophoretically resolved through 4–20% gradient gels (Bio-Rad, Hercules, CA). Antibodies used were PIAS3 (#4164; Cell Signaling Technology, Danvers, MA), STAT3 (#sc-7179, Santa Cruz Technology, Santa Cruz, CA), and *β*-Actin (#A-5441, Sigma-Aldrich, St. Louis, MO). The working antibody dilutions were: PIAS3 (1:1000), STAT3 (1:2000), *β*-actin (1:50,000). Secondary antibodies were used at 1:10,000.

### Protein quantification

Image J was used for accurate quantification of protein expression on western blots. The density profiles of PIAS3 and *β*-actin bands were obtained from x-ray films and the background subtracted out. An estimation of the PIAS3 protein level on western blots was corrected for loading discrepancies, as determined by the *β*-actin bands.

### Colony formation

Calu-1 cells were seeded in six well plates overnight and treated with 1 or 5 *μ*mol/L curcumin for 10 days. Cells were stained with 0.5% crystal violet and destained with water.

### MTS assay

Five thousand Calu-1 cells were seeded as multiple replicates in a 96-well plate and the next day treated with 5 *μ*mol/L curcumin for 72 h. Viability was measured using the Cell Titer 96 Aqueous One Solution Cell Proliferation Assay (Promega, Madison, WI) at 490 nm absorbance. The mean was calculated from replicate wells.

### Flow cytometry

Calu-1 cells were seeded in 100 mm dishes and the next day treated with 1 *μ*mol/L or 5 *μ*mol/L curcumin for 72 h. All attached and floating cells were washed with phosphate-buffered saline (PBS) and fixed in 0.125% formaldehyde followed by methanol. After incubation at −20°C, cells were washed with PBS and incubated for 45 min at 4°C in 50 *μ*g/mL propidium iodide, 0.1% Nonidet P-40, 20 *μ*g/mL RNase A, and 0.1% sodium azide in PBS. Propidium iodide fluorescence was measured on an EPICS XL-MCL cytometer (Beckman Coulter, Pasadena, CA).

### Immunohistochemistry

A MaxArray Human Lung Cancer Tissue Microarray (TMA) was obtained from Invitrogen and rinsed with xylene for deparaffinization and transferred through two changes of 100% ethanol. The TMA was treated with 2.5% hydrogen peroxide/methanol buffer to inhibit endogenous peroxidase and boiled in a pressure cooker with sodium citrate buffer. After antigen retrieval, the slide was blocked using Background Sniper (#BS966M, Biocare, Concord, CA) for 20 min and stained overnight at 4°C with rabbit anti-human PIAS3 antibody (Cell Signaling Technology) at 1:400. Anti-rabbit MACH4 horseradish peroxidase-labeled polymer secondary antibody (Biocare #MRH534L) was then applied for 30 min. The slides were rinsed in a Tris-buffered saline (TBS) series and visualized with a 10-min incubation of liquid 3,3′-diaminobenzidine in buffered substrate in the dark. Finally, the slides were counterstained with hematoxylin for 30 min and mounted with Biomount. PIAS3 immunostaining was stratified into three scoring categories: 0–1 = no or minimal uptake, 2 = moderate staining, and 3 = strong staining in >50% of tumor nuclei. This PIAS3 scoring system was developed by a thoracic pathologist.

### Statistical methods

The original lung squamous cell carcinoma level 2 gene expression data and clinical data were downloaded from the TCGA website with the platform Affymetrix HT_HG-U133A. Gene expression data and clinical data were available on a total of 133 patients. The TCGA gene expression data for PIAS3 (203035_s_at) was extracted and merged with clinical outcome data for further analysis. We examined the predictive value of PIAS3 on survival treating PIAS3 expression as a continuous measurement as well as a categorical variable, that is, by collapsing PIAS3 gene expression values into three groups: below 25% (33 patients), between 25% and 75% (66 patients) and above 75% (34 patients). The Kaplan–Meier method was used to estimate the overall survival and the difference of survival among groups was examined using a log-rank test. The Cox proportional hazards model was performed to estimate the predictive value of PIAS3 gene expression on survival, and the hazard ratio was obtained from this model. Analyses were performed using SAS version 9.3 statistical software (SAS Institute Inc, Cary, NC) or R 2.1.15.

## Results

### PIAS3 expression is low in squamous cell lung cancer

**Figure 1:**
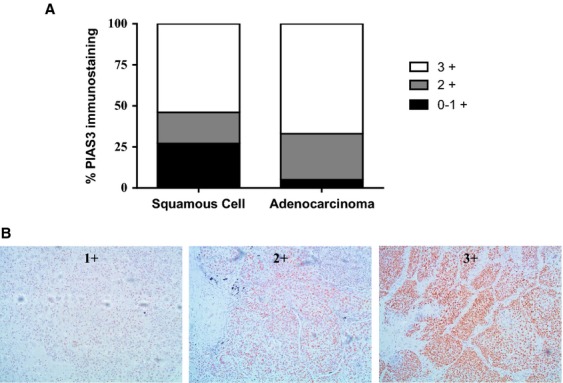
PIAS3 expression is low in squamous cell compared to adenocarcinoma of the lung by immunohistochemistry. (A) A thoracic tissue microarray was probed by immunohistochemistry with PIAS3 antibody and scored by a thoracic pathologist. The bar graph shows the percentage of tissue cores yielding the indicated IHC score for squamous cell (*N* = 25 cores) compared to adenocarcinoma (*N* = 21 cores). (B) Representative pictures of PIAS3 staining and scoring.

Previous work from our laboratory suggested differential PIAS3 expression among the principal histologic subtypes of non-small-cell lung cancer (NSCLC); namely that squamous cell carcinoma demonstrated lower PIAS3 expression than adenocarcinoma, by western blotting cell lines and immunohistochemistry of resected tumors. To validate this finding, a TMA containing a total of 60 cores of different thoracic malignancies was stained for PIAS3 expression. As shown in Figure1A, 7 of 26 total squamous lung cancer cores stained minimally (score = 0 or 1) compared to 1 of 19 adenocarcinoma cores (26.9% vs. 5.2% respectively, *P* = 0.044). These results confirm our earlier observation that PIAS3 protein expression is lower in squamous cell carcinoma compared with adenocarcinoma of the lung. Representative images of variable PIAS3 positivity in squamous lung cancer cores are depicted in Figure1B.

**Figure 2:**
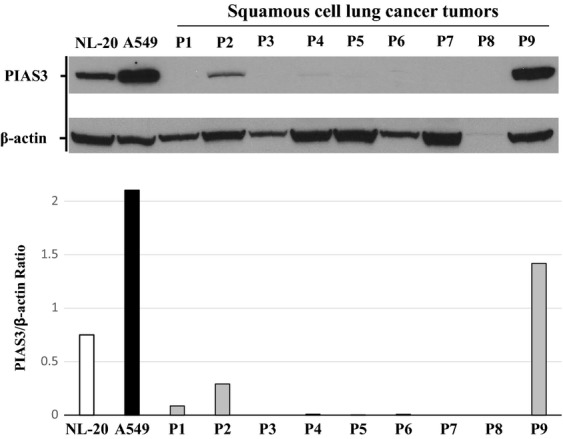
PIAS3 expression is low in the majority of squamous cell carcinoma lung tumors by western blotting. Protein precipitates of squamous cell lung cancer tumors from LCBRN were solubilized and analyzed for PIAS3 and *β*-actin expression by western blotting (top). The PIAS3 and corresponding *β*-actin bands were quantified using image J software and a ratio of the resulting values is shown (bottom). NL-20 and A549 protein lysates were used as controls.

We next used western blotting to confirm the low expression of PIAS3 protein in squamous cell lung cancer. Patient tumor tissue was obtained from the LCBRN, a publically available repository of lung cancer tissue specimens with clinical annotation that is maintained for biomedical research. Indeed, the large majority of human tumor samples showed minimal expression of PIAS3 when compared to a normal lung epithelial cell line (NL-20) and an adenocarcinoma cell line (A549) (Fig.2A). When normalized to actin, PIAS3 expression level in the squamous tumor samples was consistently lower compared to NL-20, except for one tumor sample, and even more distant from the A549 PIAS3 expression level (Fig.2B). Multiple tumor stages were included for analysis but showed no correlation with PIAS3 expression.

### Low PIAS3 transcript levels in vivo confer poor survival in squamous cell lung cancer

**Figure 3:**
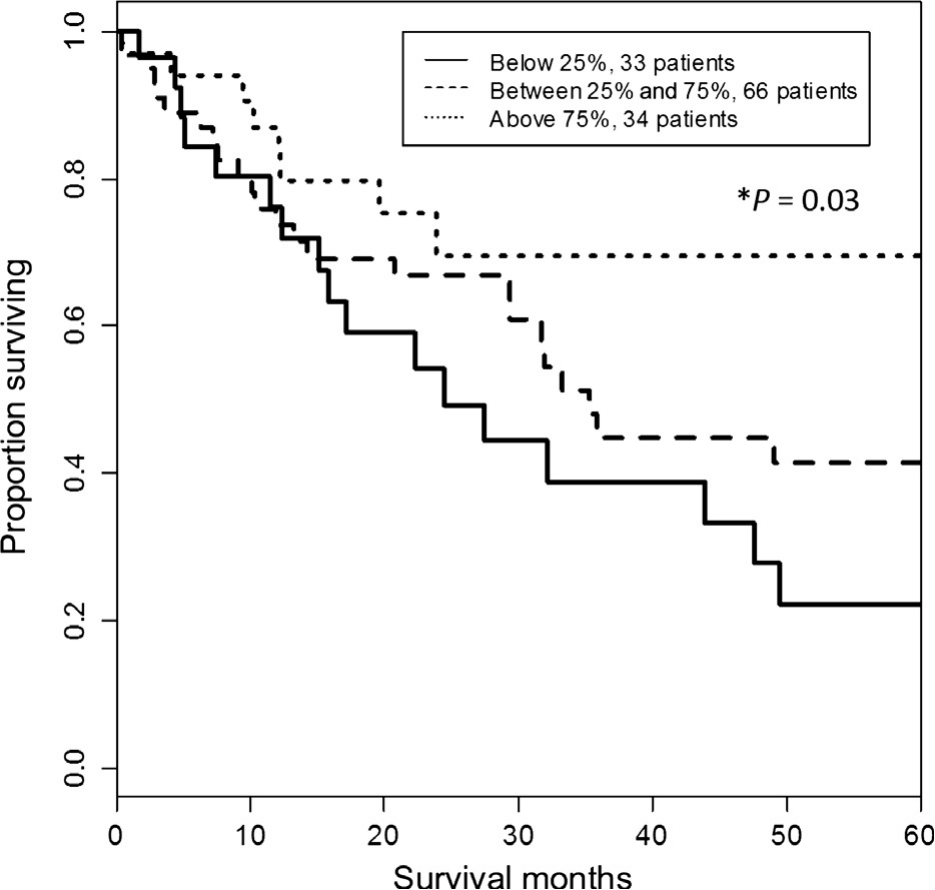
*PIAS3* mRNA transcript levels correlate with survival. *PIAS3* transcript levels and survival data were extracted from the TCGA database of squamous cell lung carcinoma. The Kaplan–Meier method was used to estimate overall survival by *PIAS3* level. A trend using three levels of *PIAS3* expression was significant, with best survival for *PIAS3* >75% and worst survival for *PIAS3* <25% (*P* = 0.03).

To investigate further the distinct PIAS3 suppression in the majority of squamous cell lung cancer specimens and its potential clinical impact, we used the TCGA database to correlate *PIAS3* mRNA transcript levels with overall survival in a cohort of 133 squamous cell lung cancer patients (Fig.3). A significant trend was found using three levels of *PIAS3* expression: <25%, 25–75%, and >75%. The best survival was associated with *PIAS3* expression >75% and the worst for *PIAS3* expression <25% (*P* = 0.03). Furthermore, Cox regression analysis revealed *PIAS3* mRNA transcript expression and patient survival demonstrated a significant correlation (Wald test *P* = 0.0092), irrespective of disease stage. As a result, a hazard ratio of 0.57 (95% CI: 0.37–0.87) was obtained, indicating a decrease in the risk of death by 43% for every 1 unit elevation in *PIAS3* gene expression. *PIAS3* transcript levels appeared, on the other hand, unaffected by tobacco use and smoking history, as measured by the number of pack years or whether the smoking was active, recent within the last 15 years or remote beyond 15 years (data not shown).

### Cultured cells reflect the low PIAS3 protein expression found in vivo

**Figure 4:**
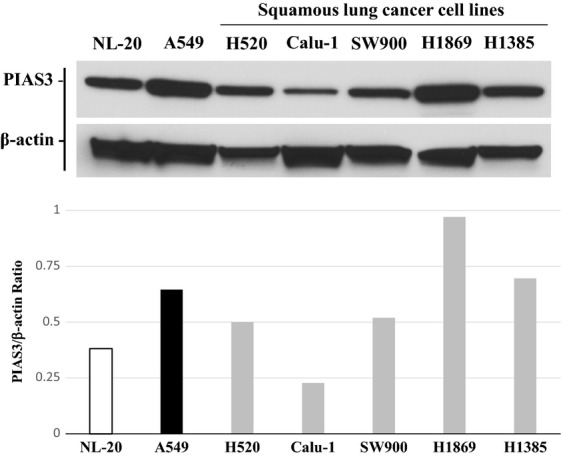
PIAS3 expression is low in most squamous cell carcinoma cell lines by western blotting. Protein precipitates of squamous cell lung cancer cell lines were prepared and analyzed for PIAS3 and *β*-actin expression by western blotting (top). The PIAS3 and corresponding *β*-actin bands were quantified using image J software and a ratio of the resulting values is shown (bottom). NL-20 and A549 protein lysates were used as controls.

To identify a model system to investigate the regulation of PIAS3 expression, we examined PIAS3 protein expression by western blotting across five squamous lung cancer cell lines (Fig.4A). As a positive control, we used the same two cell lines, NL-20 and A549, as used previously to examine tumor PIAS3 expression. Again, prominent PIAS3 protein expression was observed in A549 adenocarcinoma cells and was greater than that observed in the normal NL-20 cells. Importantly, four of five squamous carcinoma cell lines demonstrated equivalent to lower PIAS3 expression level compared to NL20 cells, and further decreased compared to the level in A549 cells (Fig.4B). From these results, we chose the Calu-1 cell line as our model system because its low PIAS3 expression best reflected the pattern observed in the human tumor specimens.

### Curcumin induces PIAS3 expression in squamous lung cancer cell lines

**Figure 5:**
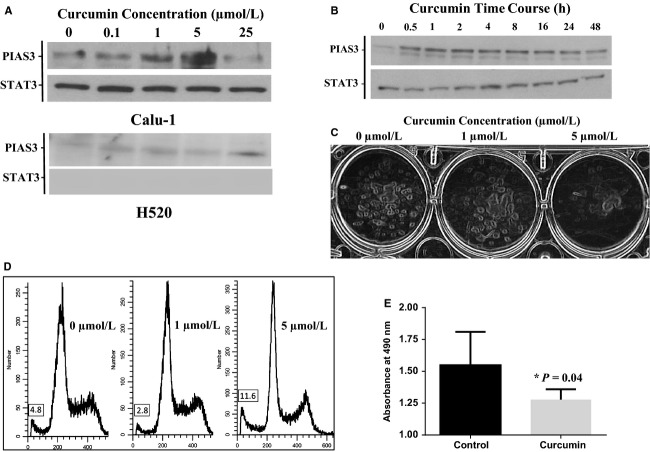
Curcumin induces endogenous PIAS3 expression and decreased cell survival in SCC cells. (A) Western blot for PIAS3 showing dose-dependent response of Calu-1 and H520 cells to curcumin treatment, in *μ*mol/L, after 24 h. (B) Western blot of PIAS3 expression showing time-dependent response of Calu-1 cells to 5 *μ*mol/L curcumin treatment, in hours. (C) Clonogenic assay demonstrating a dose response of Calu-1 cells to curcumin treatment, in *μ*mol/L, after 10 days. (D) FACS analysis demonstrating a dose response of Calu-1 cells to curcumin treatment, in *μ*mol/L, after 24 h. The percentage of cells in the subG1 phase is shown in the box. (E) MTS assay demonstrating decreased Calu-1 cell viability after incubation with 5 *μ*mol/L curcumin for 24 h.

Three squamous lung cancer cell lines (Calu-1, H520 and SW900) were treated with a concentration range of curcumin from 0.1 to 25 *μ*mol/L for 24 h. Curcumin induced a dose-dependent increase in PIAS3 expression in Calu-1 and H520 cells (Fig.5A), but not SW900 cells (data not shown). Calu-1 cells were more sensitive than H520 cells, responding maximally to a treatment with 5 μmol/L of curcumin. Further experiments showed that PIAS3 protein expression is increased as early as 30 min after curcumin treatment and remains elevated for at least 48 h in the Calu-1 cell line (Fig.5B).

### PIAS3 expression decreases cell viability of squamous lung cancer cells

To determine if curcumin induction of PIAS3 protein affects cell growth, a colony formation assay was performed and demonstrated a dose-dependent decrease in the survival of Calu-1 cells after prolonged treatment (Fig.5C). By flow cytometry, a higher proportion of Calu-1 cells accumulated in the subG1 phase in response to curcumin treatment compared to untreated controls (11.6% vs. 4.8% respectively) (Fig.5D). We further investigated the effect of curcumin on Calu-1 cell viability at the peak dose response of 5 *μ*mol/L using the MTS assay. Treatment of Calu-1 cells for 72 h with curcumin induced a significant decrease in cell viability compared to untreated control cells (1.31 vs. 1.56 respectively, *P* = 0.04) (Fig.5E). Taken together, these results demonstrate that curcumin-induced PIAS3 expression has an inhibitory effect on cell growth and survival in Calu-1 cells.

## Discussion

After long stagnation in the progress of treating metastatic squamous cell cancer of the lung, the TCGA research network finally revealed a comprehensive genomic characterization of squamous cell lung cancer 17; providing information vital for the development of personalized and potentially more effective therapy in this cancer. Squamous cell lung cancer was shown to be a complex disease entity, with a high rate of copy number alteration when compared to other malignancies such as ovarian, GBM, colorectal, breast, and renal cancer. Importantly, the data also demonstrated the potential activation of STAT3 by a number of upstream receptor tyrosine kinase signaling pathways found to be recurrently amplified in squamous cell lung carcinoma, including PDGFRA and/or KIT, EGFR, and FGFR1. Thus, the targeting of STAT3 by PIAS3 as a mechanism for tumor inhibition may represent a promising treatment in SCC.

We demonstrate for the first time that PIAS3 expression is low to negligible in a majority of SCC tumor specimens by western blotting (Fig.2). This was validated by our finding of low to negligible PIAS3 staining by immunohistochemistry in almost 50% of SCC tumor cores on a lung cancer TMA (Fig.1). Furthermore, SCC demonstrates lower expression levels of PIAS3 compared to adenocarcinoma and normal epithelium, in vitro and in vivo, by immunohistochemistry and western blotting (Fig.1, 2, and 4), consistent with our previous published data.

The clinical significance of low *PIAS3* expression was demonstrated in a large cohort of 133 squamous cell lung cancer patients registered in the TCGA database. Interestingly, we could again identify a *PIAS3* “deficient” subgroup in squamous lung cancer patients using mRNA expression levels (Fig.3). Of great importance, however, *PIAS3* transcript levels predicted survival in this patient population; higher *PIAS3* transcript levels were associated with a lower mortality at a HR of 0.57, signifying a 43% benefit in the risk of death for every unit of increase in *PIAS3* mRNA expression. Taken together, these results reveal the importance of PIAS3 as a tumor suppressor of STAT3 activity in squamous cell lung cancer.

Curcumin, a natural drug derived from the spice turmeric, has been extensively studied as an anticancer agent 18,19. It has been shown to inhibit STAT3 phosphorylation and activity in melanoma cells 20, ovarian and endometrial cancer cells 21, and small-cell lung cancer cells 22. Indeed, Calu-1 cells, a model of SCC PIAS3 deficiency, also responded to curcumin treatment with concentration-dependent PIAS3 overexpression, confirming a previous observation in ovarian and endometrial cancer cells 21. PIAS3 overexpression was associated with decreased viability and cell cycle arrest in SCC. Thus, we hypothesize that SCC inhibition may be achieved clinically by reversing the PIAS3 deficiency present in a subgroup of squamous cell tumors, restoring its inhibition of STAT3-mediated cell proliferation. Little is established, however, about the mechanism of PIAS3 downregulation in cancer. Curcumin represents a tool to explore this question and thereby promote therapeutic strategies aimed at restoring PIAS3 expression in SCC.

While curcumin has been utilized in the current setting, its pharmacologic profile is complex and its bioavailability poor 18,23. This necessitates the discovery of other small molecule activators of PIAS3 expression, perhaps a preexisting therapeutic agent, if we hope to achieve our goal of PIAS3-targeted therapy in squamous cell lung cancer. Another key area to explore is when in the process of squamous cell lung cancer development does PIAS3 expression decrease? If this decrease were to occur at the carcinoma in-situ stage, this could potentially indicate the use of agents derived from curcumin as chemo-preventative agents.

## Conflict of Interest

None declared.
